# Effects of Buried Wood on the Development of *Populus tremuloides* on Various Oil Sands Reclamation Soils

**DOI:** 10.3390/f13010042

**Published:** 2022-01-01

**Authors:** Kaitlyn E. Trepanier, Laura Manchola-Rojas, Bradley D. Pinno

**Affiliations:** Department of Renewable Resources, University of Alberta, Edmonton, AB T6G 2R3, Canada; ktrepani@ualberta.ca (K.E.T.); manchola@ualberta.ca (L.M.-R.)

**Keywords:** buried wood, land reclamation, reclamation soils, trembling aspen, tree growth

## Abstract

Buried wood is an important but understudied component of reclamation soils. We examined the impacts of buried wood amounts and species on the growth of the common reclamation tree species trembling aspen (*Populus tremuloides).* In a greenhouse study, aspen seedlings were planted into four soil types, upland derived fine forest floor-mineral mix (fFFMM), coarse forest floor-mineral mix (cFFMM), and lowland derived peat and peat-mineral mix (PMM), that were mixed with either aspen or pine wood shavings at four concentrations (0%, 10%, 20% and 50% of total volume). Height and diameter growth, chlorophyll concentration, and leaf and stem biomass were measured. Soil nutrients and chemical properties were obtained from a parallel study. Buried wood primarily represents an input of carbon to the soil, increasing the C:N ratio, reducing the soil available nitrogen and potentially reducing plant growth. Soil type had the largest impact on aspen growth with fFFMM = peat > PMM > cFFMM. Buried wood type, i.e., aspen or pine, did not have an impact on aspen development, but the amount of buried wood did. In particular, there was an interaction between wood amount and soil type with a large reduction in aspen growth with wood additions of 10% and above on the more productive soils, but no reduction on the less productive soils.

## 1. Introduction

Within Alberta, Canada, oil sands surface mining has disturbed an area of 953 km^2^ out of 4800 km^2^ of surface mineable area, and all of this area must be reclaimed once industrial operations are complete [1,2,3]. Before mining occurs, the area is cleared of merchantable timber and the remaining woody debris (ground logs, limbs, standing snags, stumps and twigs) is burnt or mulched to be used in reclamation projects, such as by incorporating it into the salvaged reclamation soils or by placing it on top of the reclamation soils [4]. Once the merchantable timber is removed, the soil is salvaged and either directly placed onto reclamation sites or stockpiled for future use. Following mining, the aim of reclamation in the oil sands is to create a self-sustaining ecosystem that has an equivalent land capability to the ecosystem that was present pre-disturbance [1,4,5,6]. A self-sustaining ecosystem includes various components such as soil, plants, and wildlife, along with ecosystem processes such as primary productivity and nutrient cycling [6,7,8]. Within the soil, buried wood has the potential to alter nutrient availability, water holding capacity and microbial communities, but its impact on tree growth in these reclamation soils is not known [9,10,11].

Buried wood is found in all forests worldwide, including those of both natural and anthropogenic origin, but its impacts on ecosystem processes such as tree growth are understudied [12,13]. Buried wood occurs naturally through catastrophic events (i.e., landslides or floods) which lead to rapid burial [14] or through individual tree falls being covered with vegetation and gradually decomposing [15]. In anthropogenic forests, such as oil sands reclamation sites, buried wood occurs mechanically with timber harvesting, reclamation cover soil salvage, placement resulting in the mixing and burial of woody material, and reclamation practices such as mulching and placement of coarse woody debris [16,17]. Buried wood is a critical factor controlling microbial communities, nutrient availability, and microsites for seedling establishment in reclamation soils, but there are currently few studies that we are aware of that focus on buried wood in oil sands reclamation soils; most of the focus has been on coarse woody debris [9,10,11].

In the Alberta oil sands region, upland reclamation soils generally consist of mixtures of organic and mineral soil components derived from either upland or lowland environments [16]. Upland derived reclamation soils include fine (fFFMM) and coarse (cFFMM) textured forest floor-mineral mix [16]. These soils are mixtures of the surface organic forest floor layer with underlying mineral soil [16]. Lowland derived reclamation soils include peat and peat-mineral mix (PMM), both of which are widely used in oil sands reclamation due to their abundance in the landscape [16]. FFMM soils, have been found to have more diverse and abundant microbial communities compared to Peat and PMM soils, which may lead to an increased rate of decomposition and subsequent nutrient release [9,16,18]. However, there has been conflicting results in comparing the two materials in terms of their ability to release nitrogen (N), with some studies showing higher N release rates for FFMM than PMM [6], while other studies demonstrate the opposite [18,19]. However, in a laboratory incubation study comparing FFMM and PMM, total N concentrations and extractable nitrate was greater in PMM than FFMM, but extractable ammonium and phosphate concentrations where higher within the FFMM [20]. These differences in potential mineralization may impact wood decomposition rates and the timescale of nutrient immobilization and subsequent release.

In terms of soil nutrient availability, buried wood is potentially both a source and a sink for nutrients depending on time scale and decomposition status. Buried wood is generally a resource of unavailable nutrients until decomposers breakdown the wood leading to an increase of nutrients available to plants including potentially an initial increase of nitrogen [10,21]. Compared to nitrogen, there is little evidence that buried wood decomposition influences the levels of phosphorus [22]. In later decomposition stages, C loss increases and nutrients are released in forms available for plant intake [23]. There is little information available on the impact on various levels of buried wood within the soil. However, with an increase in buried wood, higher amounts of nitrogen will be immobilized by microbes decomposing the wood, leaving lower levels of nitrogen for plants to have access to within the soil. Due to the differences in cellulose, hemicellulose and lignin contents found between conifer and broadleaf wood, the timelines for nutrient immobilization and mineralization may also vary [23]. For example, lignin, a complex structure resistant to decomposition, is higher in coniferous wood (25–35%) compared to broadleaf wood (18–25%) [23]. Therefore, the immediate immobilization of nutrients in response to buried wood addition may vary by wood species. In our study, we compare the impacts of coniferous (pine) and broadleaf (aspen) wood on tree growth.

Buried wood decomposition is expected to have varying effects on cFFMM, fFFMM, peat and PMM, with the largest initial impact on the FFMM soils due to their lower initial C content. The organic-based peat and PMM soils have a higher initial C content and a higher C:N ratio due to the high amount of undecomposed plant material (peat) [24,25], so adding more C in the form of buried wood may have little effect on these soils.

Trembling aspen (*Populus tremuloides* Michx.) is a common tree species found within upland boreal forests and is a desired species for establishment on land reclamation sites [26,27,28,29]. Aspen is a fast-growing, nutrient demanding species and reacts strongly to changes in environmental conditions, making it ideal for short-term experiments such as ours [26,27,28,29]. Aspen growth has been positively linked with soil nitrogen (N), potassium (K) and calcium (Ca) concentrations within the soil [28,30]. Furthermore, plant growth is strongly related to nitrogen availability since it is used to form amino acids that will be part of structural proteins and enzymes, increasing metabolic activity and cell division (growth, diameter, and biomass) [18,20,31,32]. A major part of the nitrogen in plants is utilized in the synthesis of chlorophyll, so it is also necessary for photosynthesis [33,34]. Therefore, the potential impact of buried wood on nutrient availability may have an impact on the growth and development of aspen seedlings on reclamation sites. The purpose of this study is to determine the potential impacts of buried wood type and amount within various reclamation soils on aspen seedlings.

## 2. Materials and Methods

### 2.1. Soil Characteristics

Four different soil types collected from an oil sands mine located in northern Alberta were used in this experiment. Fine forest floor-mineral mix (fFFMM) was collected from the cover soil (top 30 cm, forest floor + upper mineral soil) of a two-year-old reclamation site in late August 2016 and remained in storage until used for this experiment in May of 2020. Coarse forest floor-mineral mix (cFFMM, top 20 cm, forest floor + upper mineral soil) and peat (from the top 0.3 m to 1.3 m, organic layer) were obtained from pre-salvaged sites in May of 2020. Peat-mineral mix (PMM) was prepared in the lab using peat and underlying mineral sand; the PMM was mixed to a volumetric ratio of 60:40 peat to sand. All soil types were sieved to ensure samples were uniform and to remove large debris such as wood, rocks, and coarse roots.

Soil nutrient supply rates were determined using Plant Root Simulator (PRS^®^) probes (Western Ag Innovations, Saskatoon, SK, Canada) from an aerobic lab incubation. Probes were inserted into bags of each soil and incubated for seven days at 70% of field capacity. The probes were extracted, rinsed thoroughly with Ultrapure water, and sent to Western Ag for extraction with 0.5 M HCl and analysis with inductively-coupled plasma spectrometry (Optima 8300 ICP-OES; Perkin Elmer Inc., Woodbridge, ON, Canada) to measure ion contents; total inorganic nitrogen (TIN = NH_4_-N and NO_3_-N) was determined via colorimetry with an automated flow injection analysis system (FIAlab Instruments Inc., Bellevue, WA, USA). Field capacity was determined using a 5 Bar Ceramic Plate Extractor (SoilMoisture Equipment Corp., Santa Barbara, CA, USA) to extract the excess water of saturated soil subsamples with compressed air pressure, and field capacity was calculated with the dry and wet soil weights. Samples for measuring electrical conductivity and pH were prepared following the Kalra and Maynard method [35] with deionized water; a SevenEasy pH meter and a FiveEasy Conductivity meter (© Mettler Toledo) were used. All analyses were conducted in triplicates. Aspen and pine wood shavings were sourced from commercially used kiln-dried animal bedding.

Peat was the most acidic soil, with a pH of 4.42, as well as being the soil with the highest field capacity (194%). All other soils had near neutral pH values and field capacities from 15% to 30%. The total inorganic nitrogen (NH_4_-N + NO_3_-N) supply rate was highest in fFFMM and PMM; phosphates (H_2_PO_4_^−^, HPO_4_^2−^) had a rate of less than 1 µg/10 cm^2^/7 days in all soils except cFFMM; potassium (K+) had the highest supply rate in cFFMM (253 µg/10 cm^2^/7 days) followed by fFFMM. PMM had the highest C:N ratio of 40.54, and the rest of soils had ratios from 18.57 to 20.45 (Table 1).

### 2.2. Experimental Design

The experimental set up in the greenhouse consisted of 4 soil types (fFFMM, cFFMM, PMM and peat) × 2 species of wood shavings (aspen and pine) × 4 volumetric percentages of shavings (0, 10, 20, 50%) × 8 replicates in complete blocks. Within each block, there were 28 seedlings, 7 from each soil type (one control and three from each wood type at each percentage) for a total of 224 pots. However, 17 seedlings died across the range of treatments and were removed from the analysis.

The aspen seedlings used in this study were sowed in the spring of 2019 at the Bonnyville Forest Nursery using seed from open-pollinated natural stands. They were grown in standard styroblock containers as plug stock (plugs 12 cm in length and 4 cm diameter) for one growing season and then transplanted into our treatment pots. At the beginning of our experiment, the seedlings had an average height of 21.7 cm and a basal diameter of 2.6 mm with no significant differences among treatments (perANOVA, *p* > 0.05). The seedlings were grown inside a greenhouse for 17 weeks at 20 °C with a daily photoperiod of 16 h, spot watered with tap water when needed, and no fertilization was applied.

Measurements were taken every two weeks and seedlings were then randomly rearranged within the blocks to reduce any effects of placement. The height (cm) of seedlings was measured from week one and was measured from the base of the soil to the terminal bud of the tallest stem. Foliar chlorophyll concentration measurements started to be collected in week three and were taken on every seedling based on an average of three leaves. At the end of week 17, the leaves and stem were harvested and dried at 70 °C for 48 h while measuring plant material over two days to ensure samples were at an oven-dry state, after which dry mass was determined.

### 2.3. Statistical Analysis

All data analysis was done using R software (version R.3.1.1, R Core Team 2019). All data were tested for normality and homogeneity based on the Shapiro–Wilk normality test and residual plots. Leaf mass, stem mass, total aboveground mass, initial height, height growth, diameter growth and chlorophyll concentration had non-normal distributions; therefore, non-parametric tests were completed. For the initial soil nutrients and properties, single ANOVAs were performed followed by Tukey’s HSD post hoc test. For the analysis on the effects of soil type, wood percentage and wood type on leaf mass, stem mass, total aboveground mass, height, diameter, and chlorophyll concentration, a permutational analysis of variance (perANOVA, significance level of 0.05) using the lmPerm package (version 2.1.0) with Tukey’s HSD post hoc test was used. Wood type (pine and aspen shavings) was not significant (*p* > 0.33) for any response variable, therefore it was not used in the full analysis. Pearson correlation coefficients were calculated to test the strength and direction of the relationship between chlorophyll and nutrient content and leaf:stem ratio and height. For visualization purposes, data points that were two orders of magnitude greater were excluded, but no data was excluded from the analyses.

## 3. Results

**Figure 1:**
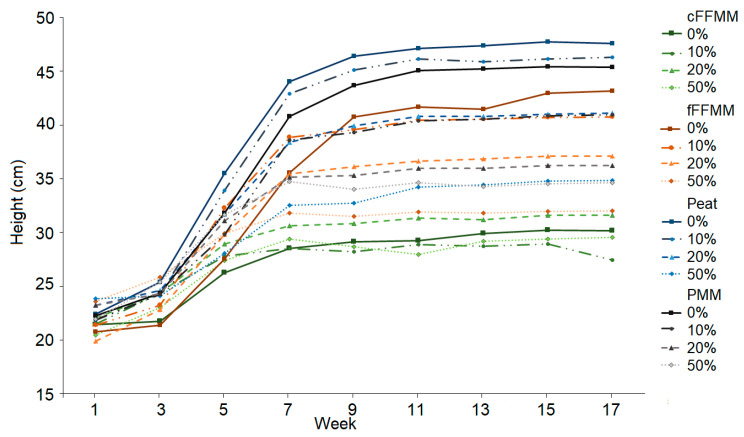
Height (cm) over time for each soil type and 0%, 10%, 20% and 50% wood amount. Values are averages. cFFMM, coarse forest floor-mineral mix (green); fFFMM, fine forest floor-mineral mix (orange); PMM, peat-mineral mix (grey); peat (blue).

**Figure 2:**
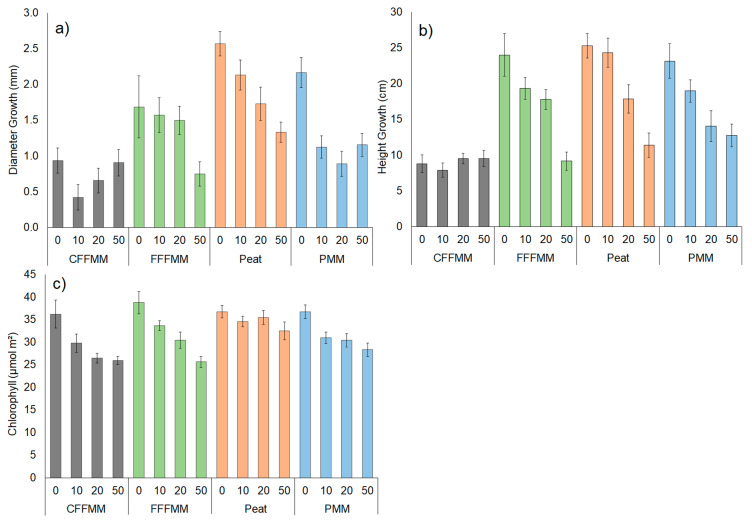
(**a**) Diameter growth (mm), (**b**) height growth (cm), (**c**) foliar chlorophyll concentration (µmol m^2^), for each soil type and 0%, 10%, 20% and 50% wood amount. Values are averages with standard error. cFFMM (grey), coarse forest floor-mineral mix; fFFMM (green), fine forest floor-mineral mix; peat (orange); and PMM (blue), peat-mineral mix.

There was a clear distinction in height among soil types (*p* < 0.001, Figure 1) representing differences in inherent productivity. Based on the height of control (0% wood) trees, peat (average = 47.6 cm at end of experiment) produced the tallest seedlings, followed by PMM (45.4 cm), then fFFMM (43.2 cm), and finally cFFMM (30.2 cm). The same pattern held for diameter, with peat having the largest diameter (average = 0.46 cm), followed by fFFMM (0.43 cm), then PMM (0.41 cm), and finally cFFMM (0.35 cm). With the addition of wood there was a decrease in height growth and diameter growth on peat, fFFMM and PMM (all *p* < 0.001); however, there was no impact on cFFMM (*p* = 0.752, *p* = 0.115) (Figure 2).

Foliar chlorophyll concentration was greatest in seedlings grown in peat, followed by fFFMM, PMM and cFFMM with concentrations similar among fFFMM, PMM and cFFMM (*p* > 0.071). Chlorophyll concentrations decreased with the addition of wood on cFFMM, fFFMM and PMM (*p* < 0.001), but on peat there was no change in chlorophyll concentrations with wood addition (*p* = 0.258) (Figure 2c).

**Figure 3:**
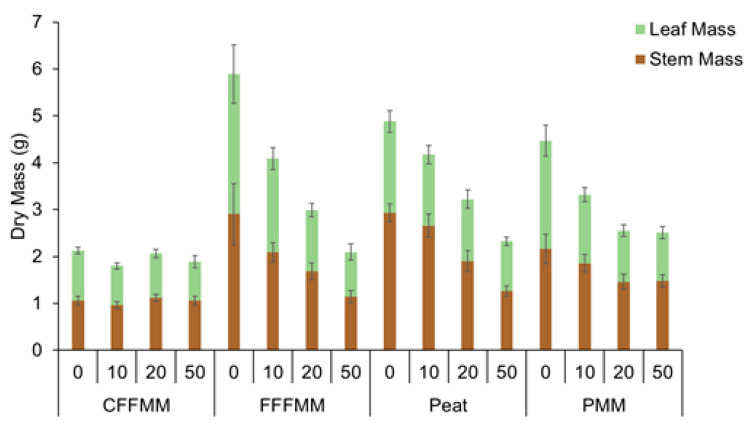
Dry leaf (green) and stem (brown) mass (g) for each soil type and 0%, 10%, 20% and 50% wood amount. Values are averages with standard error. cFFMM, coarse forest floor-mineral mix; fFFMM, fine forest floor-mineral mix; PMM, peat-mineral mix.

Total aboveground biomass for the control trees (0% wood) grown in each soil type was 5.89 g for fFFMM, 4.88 g for peat, 4.47 g for PMM, and 2.13 g for cFFMM (Figure 3) (*p* < 0.001). Seedling biomass decreased with wood addition, but the impact varied by soil type (soil type × wood interaction *p* < 0.001) such that the most productive soils had the biggest drop in growth (*p* < 0.001), while the lowest productivity cFFMM soil showed no differences in tree growth with wood additions (*p* > 0.229) (Figure 2 and Figure 3). Based on total biomass, fFFMM showed a decrease of 3.80 g from 0% to 50% wood addition (*p* < 0.001), peat showed a decrease of 2.55 g from 0% to 50% (*p* < 0.001), PMM showed a decrease of 1.95 g from 0% to 50% (*p* < 0.001), and cFFMM had no significant decrease in biomass from 0% to 50% (*p* = 0.229).

**Figure 4:**
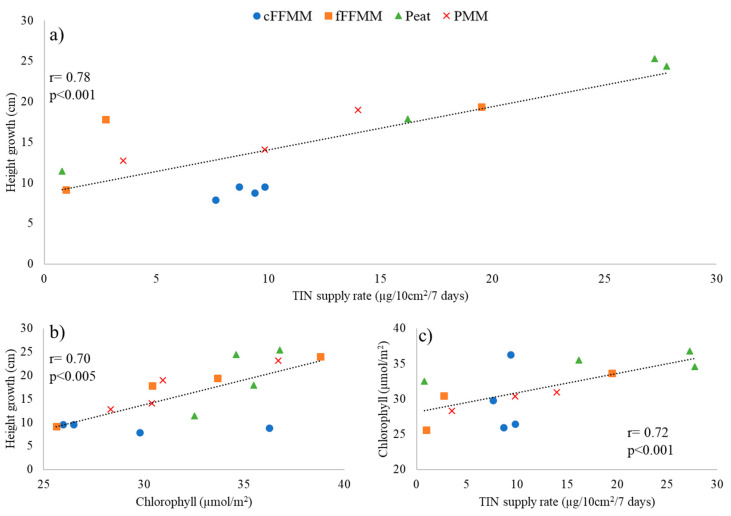
Scatter plots between height growth and total inorganic nitrogen (TIN) supply rates (**a**) (r = 0.77, *p* < 0.001), height growth and chlorophyll concentration (**b**) (r = 0.70, *p* = 0.004), and chlorophyll concentration and TIN supply rates (**c**) (r = 0.72, *p* < 0.001) among the different soil types. cFFMM (blue dot), fFFMM (orange square), peat (green triangle) and PMM (red cross). Each data point represents an average of each treatment.

Across all treatments, height growth was positively correlated to soil TIN supply rates (r = 0.78, *p* < 0.001) and foliar chlorophyll concentrations (r = 0.70, *p* = 0.004, Figure 4). Chlorophyll concentration, in turn, was correlated with soil TIN supply rates (r = 0.72, *p* = 0.002, Figure 4c). Chlorophyll concentration was also positively correlated with phosphates supply rates in cFFMM and fFFMM (*p* < 0.001), and negatively correlated with K supply rates in cFFMM and fFFMM (*p* < 0.001).

## 4. Discussion

Wood addition to the soil resulted in lower tree growth with the greatest reduction on the higher productivity soils and no impact on the lower productivity soils. In general, tree growth, diameter, biomass, and foliar chlorophyll concentration were greatest in the controls with no wood addition, and all were reduced with wood addition. Buried wood primarily represents an input of carbon to the soil, resulting in an increase in the soil C:N ratio and subsequent nitrogen immobilization [36,37]. The result is a decrease in tree biomass production as the amount of nitrogen available to the seedlings for photosynthetic activity (leaf area and chlorophyll) and subsequent growth (height, diameter, and biomass) decreases. These results coincide with studies that have found that tree height, DBH and biomass are negatively impacted when there is an increase in the soil C:N ratio [38,39,40].

There was no difference in soil nutrients or tree growth responses between aspen and pine wood additions. This could be mainly due to the disturbed lignin barrier in the wood shavings with a physical disturbance like processing the wood into shavings, or grinding by insect borers in a natural context, disrupting the lignin walls and exposing the cellulose and hemicellulose to decomposition [41]. Thus, there was no difference in resistance to breakdown between aspen and pine wood and a similar soil nutrient response. Another explanation could be that hemicellulose breakdown precedes lignin decomposition by white-rot fungi [42], so the lignin resistance is observed in a later decay stage, again resulting in no difference among wood type in this short-term experiment.

Tree productivity (height growth, biomass, diameter, and chlorophyll concentration) varied among soil types, with greatest growth in fFFMM, followed by PMM and peat. These higher productivity soils had greater declines with wood addition compared to the lowest productivity cFFMM. Nitrogen reduction, such as that which occurs after wood addition, results in a decrease in growth due to a reduction in chlorophyll concentration and photosynthetic activity [34,43]. These responses suggest that the decrease in available nitrogen in response to wood addition is the main factor driving aspen productivity. This variation in N availability with wood additions can explain the tree responses by soil. For example, fFFMM had the greatest productivity without wood and the greatest decrease with wood addition because this soil has the highest nitrogen availability and a lower C:N ratio compared to other soils used in reclamation like PMM and peat [18,44].Thus, this soil had the greatest alteration in its C:N ratio and consequently the most distinct impact on tree productivity. PMM and peat have a higher C:N ratio due to a higher proportion of organic matter and recalcitrant carbon [45] and have less nutrient availability in comparison to FFMM soils [18,20]. Therefore, the increase in the C:N ratio was not as great since these soils already had a higher amount of carbon, but still was significant enough to reduce tree growth. Finally, wood addition did not impact cFFMM because this soil was initially providing a lower productivity environment for aspen growth, evidenced by the lowest tree productivity even without wood addition. cFFMM is a sandy-textured soil, with low nutrient availability [46,47,48,49] and a low cation exchange capacity (CEC) [50]. As observed in the soils nutrients, cFFMM has a TIN supply rate 76 times lower than fFFMM, a soil high in clay, water-holding capacity, and CEC due to the surface area, structure, and surface negative charges of this mineral [51]. These results support our initial hypotheses of wood addition impacting aspen growth and having a greater impact on fFFMM than on PMM and peat; however, cFFMM was not impacted as expected. PMM is considered a high-performance soil and is used widely in reclamation practices in comparison to peat, but in this study both soils had similar responses. This could be due to the mineral component of PMM being sand and, as in cFFMM, it provides a low water and nutrient retention capacity to the soil. In contrast, if the mineral component were fine-textured with clay content, PMM could have had higher water and nutrient retention capacity, and the performance and response to buried wood may have been similar to the fFFMM.

## 5. Management Implications

This study supports the hypothesis that wood addition of 10% and above can reduce aspen growth due to an increase in the soil C:N ratio and subsequent N immobilization, which can be detrimental for tree productivity. Different species have different requirements in terms of nutrient availability, thus productivity may vary depending on the tree species. However, this study provides evidence that soils with lower total C content are more susceptible to nitrogen immobilization as a response to wood addition, and this can reduce tree growth.

It is important to keep in mind that the findings of this greenhouse study are subject to several limitations. First, the buried wood amounts used in this study are extreme values that do not necessarily represent field applications. Operationally, buried wood that is salvaged with upland and peat mineral soil is approximately a volume of 1.5% [52]. Second, this study used wood shavings, but in the field the wood is coarse-mulched, which could cause a different response. Lastly, this was a short-term study, so the detrimental effects on tree productivity observed are only in the short-term after wood application. It is outside of the scope of this study to determine long-term impact on tree growth, but it would be expected that, after wood decomposition, the immobilized nitrogen will be released to the soil and be available for plant uptake. Moving forward, some of the remaining unknowns related to the impact of buried wood in reclamation are the amount of buried wood actually found in reclamation soils and the impact this could have on field available nutrients and plant growth.

## Figures and Tables

**Table 1:** Soil nutrients and chemical properties. Values are mean and standard error. Letters indicate similarities among soils (*p* > 0.05). Total inorganic nitrogen, TIN; total carbon, TC; total nitrogen, TN; total organic carbon, TOC. Soil types: coarse forest floor-mineral mix, cFFMM; fine forest floor-mineral mix, fFFMM; peat; peat-mineral mix, PMM.

Soil Type	TIN Supply Rate (µg/10 cm^2^/7 Days)	Phosphates Supply Rate (µg/10 cm^2^/7 Days)	K Supply Rate (µg/10 cm^2^/7 Days)	pH	Electrical Conductivity (µS/cm)	FC (%)	TN (*w/w*%)	TOC (*w/w*%)	**C:N Ratio**
cFFMM	d 9.39 (1.21)	a 29.92 (3.54)	a 253.31 (61.52)	b 6.32 (0.11)	d 161.60 (0.43)	c 15.11 (0.88)	b 0.23 (0.14)	c 4.27 (0.40)	18.57
fFFMM	a 718.23 (39.40)	b 0.72 (0.07)	b 14.18 (7.08)	ab 6.54 (0.49)	c 641.0 (0.0)	b 26.91 (0.32)	b 0.18 (0.14)	c 3.55 (0.40)	19.72
Peat	c 27.25 (1.10)	c 0.38 (0.14)	c 2.62 (0.15)	c 4.42 (0.014)	a 1221.0 (2.16)	a 194.38 (1.64)	a 2.31 (0.14)	a 47.25 (0.40)	20.45
PMM	b 232.82 (66.91)	bc 0.51 (0.32)	b 7.47 (2.93)	a 6.84 (0.024)	b 764.67 (1.25)	b 30.47 (8.89)	b 0.22 (0.14)	b 8.92 (0.40)	40.54

## Data Availability

Data available upon request.
